# Surgical treatments of bone marrow lesions

**DOI:** 10.1002/jeo2.70249

**Published:** 2026-09-25

**Authors:** Luca Andriolo, Alessandro Sangiorgio, Mats Brittberg, Vincenzo Condello, Jack Farr, Elizaveta Kon, Andrew S. Levy, Giuseppe Filardo

**Affiliations:** ^1^ Clinica Ortopedica e Traumatologica 2 IRCCS Istituto Ortopedico Rizzoli Bologna Italy; ^2^ Service of Orthopaedics and Traumatology Department of Surgery, EOC Lugano Switzerland; ^3^ Cartilage Research Unit, Department of Orthopedics, Institution of Clinical Sciences University of Gothenburg and Team Orthopedic Research, Cartibase Varberg Sweden; ^4^ Joint Preservation and Reconstructive Surgery and Sports Medicine Unit Humanitas Castelli Clinic Bergamo Italy; ^5^ Jack Farr Consulting LLC Bargersville Indiana USA; ^6^ IRCCS Humanitas Research Hospital Rozzano Italy; ^7^ Department of Biomedical Sciences Humanitas University Pieve Emanuele Milan Italy; ^8^ Center for Advanced Sports Medicine Knee and Shoulder Millburn New Jersey USA; ^9^ Faculty of Biomedical Sciences Università della Svizzera Italiana Lugano Switzerland

**Keywords:** bone marrow lesions, knee, subchondral bone, surgical treatment

## Abstract

Bone marrow lesions (BMLs) of the knee are a common magnetic resonance imaging finding, defined as an alteration of the bone marrow detectable as a high signal on fluid‐sensitive sequences. A wide range of pathologies can present with BMLs, including traumatic lesions, osteoarthritis, subchondral insufficiency fractures and osteonecrosis. Several treatment options have been proposed to address BML, from conservative to surgical approaches. If nonoperative treatments fail, operative treatments are indicated. These procedures include intraosseous injections, unloading procedures and meniscus implants. In detail, intraosseous injections may be performed with biomaterials like calcium phosphate, or with orthobiologics like platelet‐rich plasma or mesenchymal stromal cells. Aim of these interventions is either to stabilise the microtrabecular tissue or to promote a healing response. Good results have been reported in the literature for many of the available surgical options, but the level of evidence is still suboptimal, and the best approach has not yet been identified.

**Level of Evidence**: Level V, expert opinion.

**Abbreviations**
ACL: anterior cruciate ligament
BMC: bone marrow concentrate
BML: bone marrow lesion
KOOS: Knee injury and Osteoarthritis Outcome Score
MRI: magnetic resonance imaging
OA: osteoarthritis
PCL: posterior cruciate ligament
PCU: polycarbonate‐urethane
UHMWPE: ultra‐high‐molecular‐weight‐polyethylene
VAS: visual analogic scale

## INTRODUCTION

Bone marrow lesions (BMLs) represent a magnetic resonance imaging (MRI) finding, which is defined as an alteration of the bone marrow and is detectable as a high signal on fluid‐sensitive sequences [35]. BMLs can originate from the subchondral or the non‐subchondral bone. A wide range of pathologies can present with BMLs, including traumatic lesions, osteoarthritis (OA), subchondral insufficiency fractures [59] and osteonecrosis [2, 61]. Microscopic trabecular fractures may be associated with macrotrauma, for example, in case of patellar dislocations or impaction injuries associated with the tibiofemoral subluxation associated with an anterior cruciate ligament (ACL) tear [31, 38]. On the other hand, repeated microtrauma may result in trabecular fractures, as in the case of spontaneous osteonecrosis of the knee (SIFK), a peculiar type of stress fracture which may or not extend to the cortical bone [55]. Nevertheless, it is also important to consider that microtrabecular fractures can be associated with altered loads of bone, ranging from loss of meniscal function to loss of the protective function of articular cartilage. Finally, ischaemic events can also be involved, with altered vascular permeability and hypoxia due to venous stasis, which represents key factors in BML onset. Additionally, ischaemic necrosis can be a consequence of the oedema itself. Several treatment options have been proposed to address BML, from conservative to surgical approaches. If nonoperative treatment fails and the patient continues to experience BML‐related symptoms despite the use of available conservative treatments, operative treatments are indicated. These procedures may be applied in several types of BML, both in younger and elderly patients. Lesions candidates for surgery will be discussed in this article.

## THE ROLE OF SUBCHONDRAL BONE

**Figure 1:**
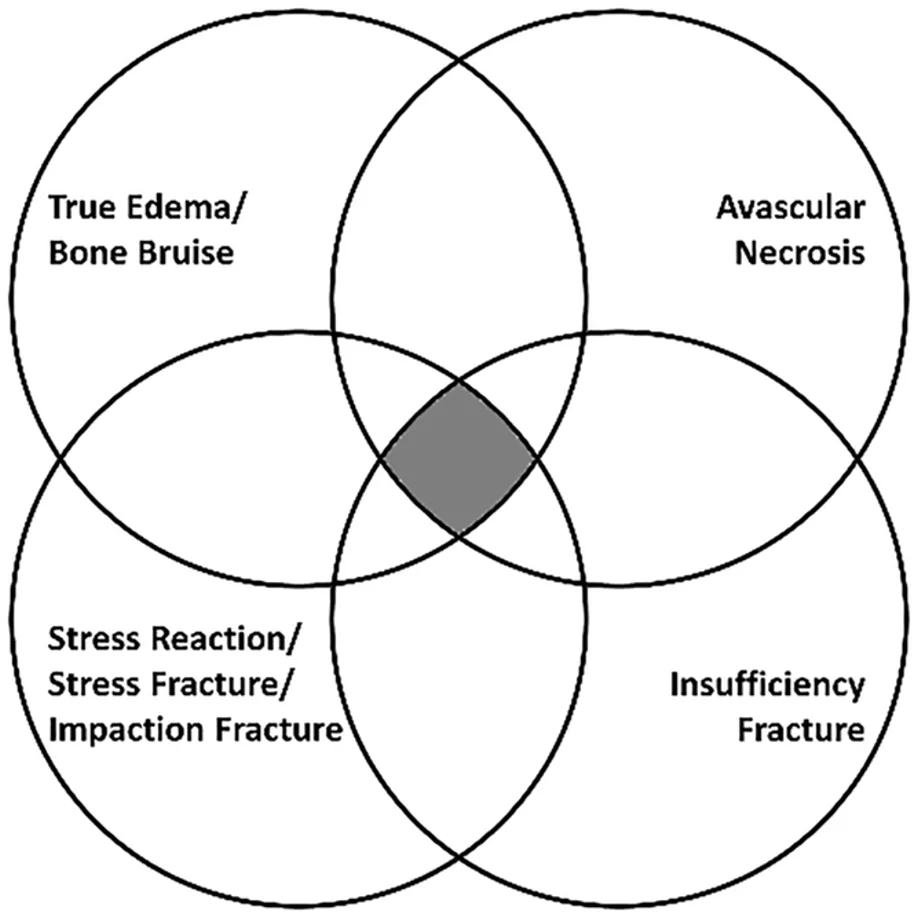
Subcategories and overlap of bone marrow lesions aetiology.

BMLs observed on T2 fat‐suppressed, or similar MRI protocols present varying underlying causes. The aetiology may range from bone bruise/true oedema to stress fractures, from insufficiency fractures to avascular necrosis (Figure 1). Regardless of the initiating factor, cartilage damage and joint derangement progression are sustained by common histopathological changes, including microcracks, micro‐oedema, microbleeding within the subchondral region, osteocyte necrosis and empty lacunae, haemorrhage and oedema, thickness of the subarticular trabeculae and subchondral bone cysts. In this context, the role of subchondral bone in the pathogenesis of cartilage damage has been underestimated in the context of cartilage repair procedures [58]. Injury to the subchondral micro‐architecture may cause abnormal stress on the cartilage layer, resulting in inadequate support from the underlying bone layer during loading [52]. Additionally, the stiffening of the subchondral plate, with increasing necrotic tissue, would favour horizontal splits in the overlying cartilage [43].

Subchondral bone is not only an important shock absorber, but it is also important for cartilage metabolism [29, 51]. The subchondral region is highly vascularised [37], and it comes in contact with the deepest hyaline cartilage layer with its terminal vessels. The perfusion of these vessels accounts for more than 50% of the glucose, oxygen, and water requirements of cartilage [29]. Bone is present in large masses, absorbing much of the forces sustained during impulsive loading and, by that, sparing the cartilage layer [29]. The osteochondral unit is therefore important and needs to be kept intact and reconstructed when damaged. Furthermore, the bone should be strong and elastic yet deformable. These aspects are crucial when performing cartilage repair. An increase in subchondral bone stiffness with a decrease in its capacity to attenuate peak dynamic forces, which act in the form of impulsive loads, could initiate damage and subsequent OA development [50]. A growing understanding of subchondral bone contribution to joint health highlighted the interconnected nature of the subchondral bone and the articular cartilage. Hwang et al. documented an augmented mutual exchange of chemicals and fluids between cartilage and subchondral bone in the course of OA [28]. This cross‐talk may be caused by changes to the microstructure of subchondral bone secondary to localised stresses, which include an increase in microcracks and microfractures of the trabeculae [6]. Finally, it has been suggested that the flow of growth factors via communicating channels during remodelling and repair of subchondral bone may initiate a feedback loop between cartilage and bone that leads to OA progression [36]. These mechanisms could also influence, besides the disease progression, the clinical manifestation. In the case of OA, for example, some authors have reported that patients presenting with BMLs were more likely to be symptomatic [18].

**Figure 2:**
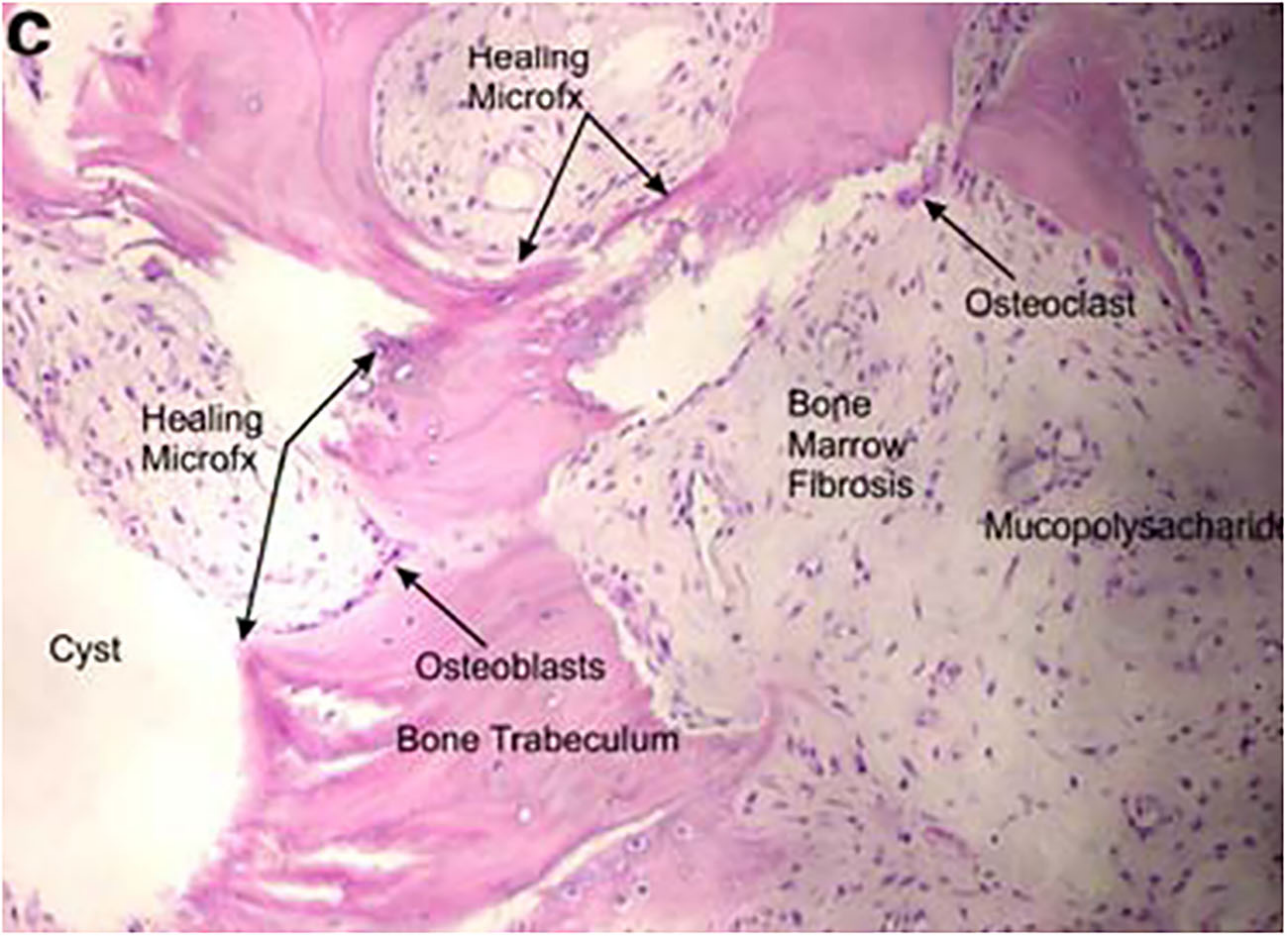
Histopathology of bone marrow lesions (BMLs) [70]. Minimal true oedema is seen, abnormal bone spicules with excessive fibrosis, small areas of osteonecrosis and extensive bone remodelling with reverse lines.

The importance of the subchondral bone in relation to chondral injuries and OA development was suggested by Radin and Rose [52]. In OA knees, a direct correlation has been documented between subchondral bone marrow oedema and patient pain as well as arthritic progression [18]. Biopsies and MRIs analysing subchondral BMLs in case of OA chondral lesions suggest the concomitant presence of interstitial oedema, fibrosis, bone remodelling and even small areas of osteonecrosis [64] (Figure 2). All these pathological features act synergically in worsening joint degeneration, with a statistically significant association between BML extent and cartilage loss [32]. When patients with pain associated with MRI signs of BML have failed conservative treatment, surgery is indicated.

## INTRAOSSEOUS BIOMATERIAL INJECTIONS—SUBCHONDROPLASTY

The goal of surgical treatments with intraosseous injections is to restore the subchondral architecture, while preventing joint degeneration. Intraosseous injections are commonly employed to address BMLs associated with stress fracture, and they may be divided into two major categories: those providing structural support (biomaterials) and those with a biologic activity (cell‐related). In the case of biomaterial structural support, many variants of calcium salts have been formulated for injection into the area of injury. The structural support provided by these crystal ceramics (composed most commonly of calcium and phosphate) is mediated by an endothermic/isothermic reaction. The ultimate goal of biomaterial injections, in addition to pain relief, is to promote the short‐term formation of moduli similar to cancellous bone, which will be replaced in the long term by healthy cancellous bone through creeping substitution. Sharkey et al. reported symptomatic improvement in patients with OA with this application modality [62]. Indications for biomaterial injections were subsequently expanded to treat even lesions associated with focal defects or in association with cartilage restoration procedures [17]. Published studies are limited to small case series, but the technology appears promising, with a recent meta‐analysis by Astur et al. reporting a 70% delay in total knee arthroplasty at 2 years [3] following calcium phosphate injections. Nonetheless, caveats remain. First, calcium salts available on the market are different. Great differences exist in flow characteristics, crystal formation and porosity, which are important for both remodelling and elasticity. The use of these calcium phosphate injections remains a promising technique that awaits randomised control trial evaluation as well a more precise characterisation of their use, even in conjunction with biologic products.

**Figure 3:**
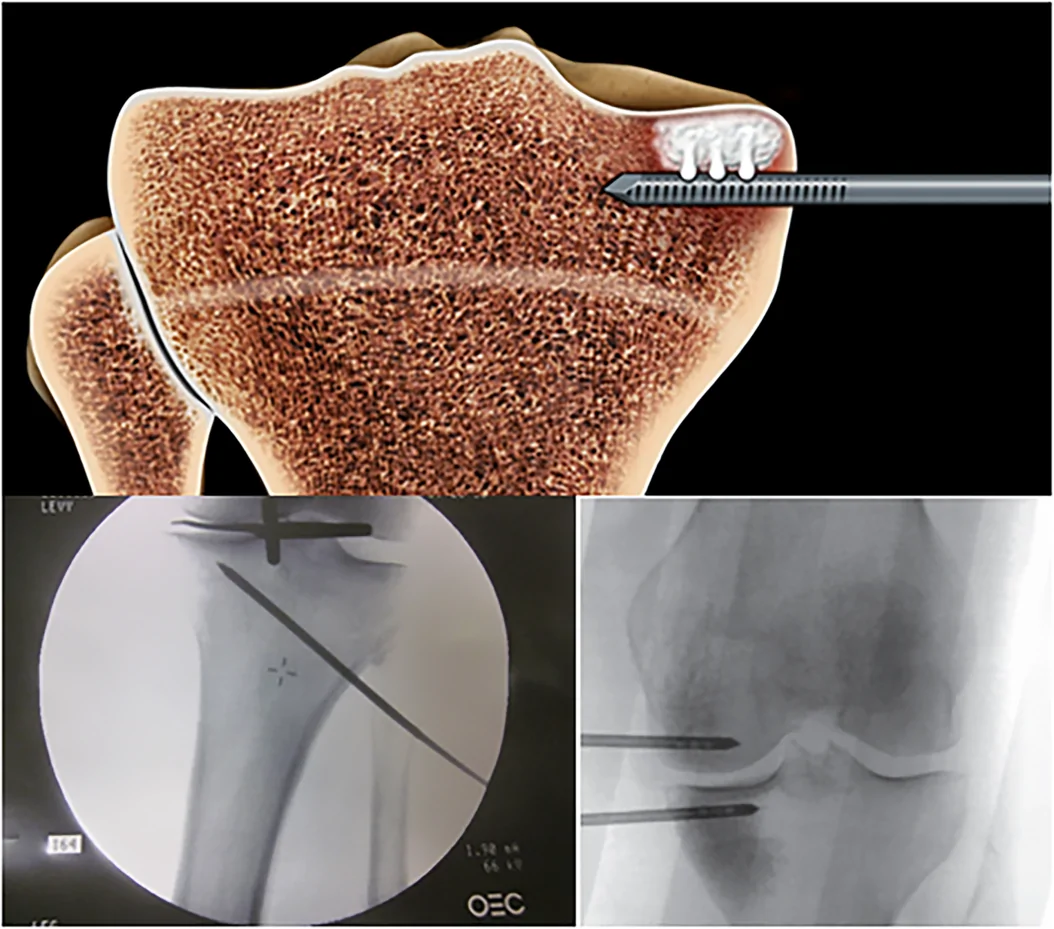
Schematic and radiographic representation of the subchondroplasty procedure.

The rationale for subchondroplasty treatment is based on considering BMLs as non‐union trabecular fractures. Subchondroplasty aims at relieving pain, slowing the progression of subchondral bone atrophy, and preventing additional chondral deterioration. The procedure consists of a localised injection of calcium pyrophosphate into the BML area [9] (Figure 3). By providing a biologic internal fixation, the mechanical enhancement of chronically damaged and structurally compromised subchondral bone can be stabilised. Calcium phosphate has been shown to be successful in fracture treatment for tibial plateau and distal radius fractures [4]. Those fracture patterns demonstrated a consistent ability to decrease pain, maintain a good reduction and facilitate an early return to pre‐injury function [57]. One of the most employed products available on the market is AccuFill® (Zimmer Biomet), an osteoconductive nanocrystalline calcium phosphate. It is injectable and does not require a bone void to be injected. It flows around bone trabeculae and endothermically hardens, ultimately gaining a compressive strength similar to cancellous bone. It is highly porous, thus allowing more surface area for revascularization and cell‐mediated remodelling. Subchondroplasty is performed in the operating room under general or spinal anaesthesia. The technique is generally performed under fluoroscopy and involves triangulation of 2.5 mm cannulas to match the BML pattern on MRI. Most commonly, it is combined with an arthroscopy to check possible leakage of the material inside the joint and to perform other associated procedures.

Initial short‐term investigations reported a marked reduction in pain visual analogic scale (VAS) scores in 85% of patients with end‐stage OA at 1 year and 70% survivorship at 2 years [10]. The same authors demonstrated that prior subchondroplasty did not affect future total knee replacement incidence and clinical results [68]. Multiple subsequent studies in non‐workers compensation patients with OA have demonstrated 70%–84% avoidance of TKA at up to 3 years with significant improvement in Western Ontario and McMaster Universities Osteoarthritis Index and Knee injury and Osteoarthritis Outcome Score (KOOS) scores [7, 27, 53]. Surprisingly, subchondroplasty has demonstrated similar early results in pain and function and long‐term survivorship in workers' compensation patients that is maintained for up to 7 years [39, 53].

Overall, subchondroplasty showed a disease‐modifying effect, with a lower risk of conversion to arthroplasty compared to other injectable treatments [56]. Long‐term and more detailed follow‐ups are needed to further evaluate the effects on overall OA progression. There is less evidence to support the use of subchondral calcium phosphate injections in cases with intact cartilage. This suggests that making the implant active with material, such as bone marrow aspirate concentrate, may be warranted.

## INTRAOSSEOUS ORTHOBIOLOGICS INJECTIONS

Treatment of osteonecrosis of the femoral head has typically involved core decompression. More recent trends may involve the use of bone marrow concentrate (BMC) as augmentation of core decompression, and in the subchondral bone pathology of the femoral head, this combined treatment has shown promising results in the past few years [69]. Moreover, randomised trials were also performed, showing how core decompression augmented with BMC improved bone healing rates when compared to core decompression alone [26]. Borrowing the rationale of combined treatment in the hip and the knee joint, the use of an intraosseous injection of orthobiologic has been investigated.

Following promising studies on the injection of orthobiologics in the area of BMLs [12, 45], the injection modalities (intra‐articular and subchondral) have been addressed, with BMC being one of the most investigated products. Good results were documented for the combined intra‐articular and subchondral bone BMC injections [34]. In particular, a significant reduction in bone marrow oedema extent was documented in symptomatic knee OA patients treated with a combination of intra‐articular and subchondral BMC injections (femoral condyle and tibial plateau) [33]. In a prospective randomised trial, 60 patients with bilateral knee OA received one intra‐articular BMC injection in one knee and subchondral BMC injection in the contralateral knee [25]. At 15‐year follow‐up, the subchondral injected knees showed significantly higher clinical and radiological improvements, with even a significant effectiveness in postponing total knee arthroplasty. Furthermore, a literature review confirmed that the application of autologous BMC in combination with scaffolds or in injection in the subchondral bone was superior to intra‐articular injections for long‐term results in treating knee cartilage lesions [24].

Nevertheless, while there is a growing body of evidence supporting the use of BMCs in the treatment of knee OA, especially in the early stages, other aetiologies of BMLs are less studied. In addition, some studies documented a tendency of stem cells to differentiate into fibrocartilage, negatively affecting the clinical outcomes after BMC injections, which should be further investigated [24]. Future research should address intralesional stem cell injection results by different pathologies in order to understand the real potential of a targeted treatment for every etiopathological presentation of BMLs.

## UNLOADING PROCEDURES

Unloading procedures represent a valuable approach to prevent joint derangement progression. The literature highlights their use mainly in the treatment of cartilage lesions in the context of OA [5, 67]. However, by reducing load on the OA compartment, a reduction of OA‐related oedema may be achieved, thus supporting the application of unloading procedures also in the treatment of BMLs.

**Figure 4:**
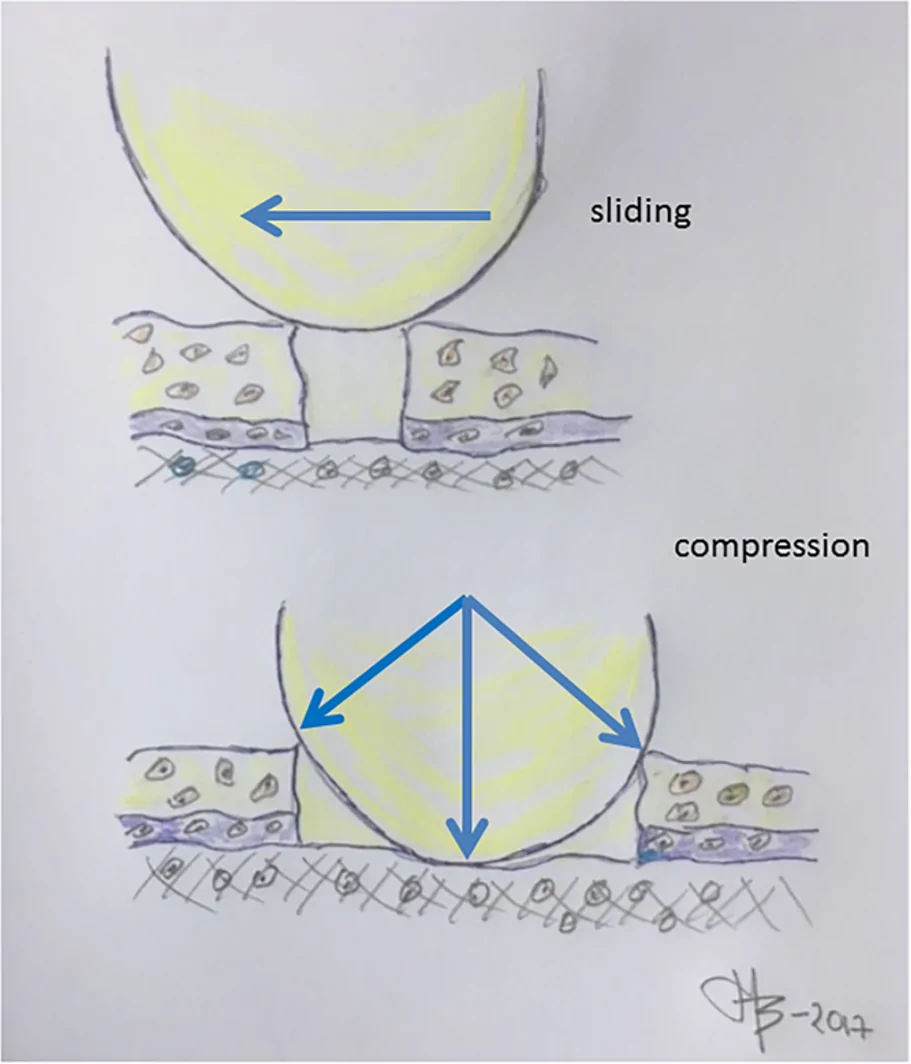
Drawing of the load characteristics in small and large cartilage defects. Small defects will mostly not be influenced by opposite loads as the opposing surface will glide/slide over the defect area. In a large defect, the opposing surface will reach the bottom of the defect and compress the edges of the defect with negative biomechanical influences and risk of lesion progression.

The proper amount of stress placed on an osteochondral graft for cartilage repair varies based on anatomical region and overall stability of the bone. Resorption may occur in areas where the bone is not exposed to the right type of mechanical stresses [66]. Thus, the weight‐bearing characteristics of each patient must be taken into account. Different cartilage lesion sizes are also influenced by the loading characteristics. In a small lesion, the loading will be on the surrounding native cartilage, while in case of a large defect, the load influences both the centre of the defect as well as the edges (Figure 4). Cartilage defects that have been successfully repaired may deteriorate after some years. This occurs more often in case of large defects, poorly contained lesions where a continuous high load may induce a progressive matrix loss. In order to protect and facilitate cartilage repair, certain parameters need to be taken into consideration and may represent an indication for unloading: lesion size, alignment, instability and weight. Temporary unloading could be achieved with crutches or with an unloader brace, while definitive unloading is done with an unloading osteotomy.

When addressing unloading surgical approaches, it is important to highlight an important concept: the following procedures do not target BML itself, as BML often resolves naturally within a few months [46]. Nonetheless, the underlying BML causes may lead to progressive joint derangement and permanent disability. In this context, BML severity is often a marker for joint pathology and rapid disease progression, warranting interventions aimed at halting or mitigating structural damage [60, 65]. Thus, the rationale for these surgeries is to treat the underlying pathologies which might contribute to the symptoms or the slow disease resolution, extending beyond the temporary presence of BML, to address long‐term joint health and functional outcomes.

**Figure 5:**
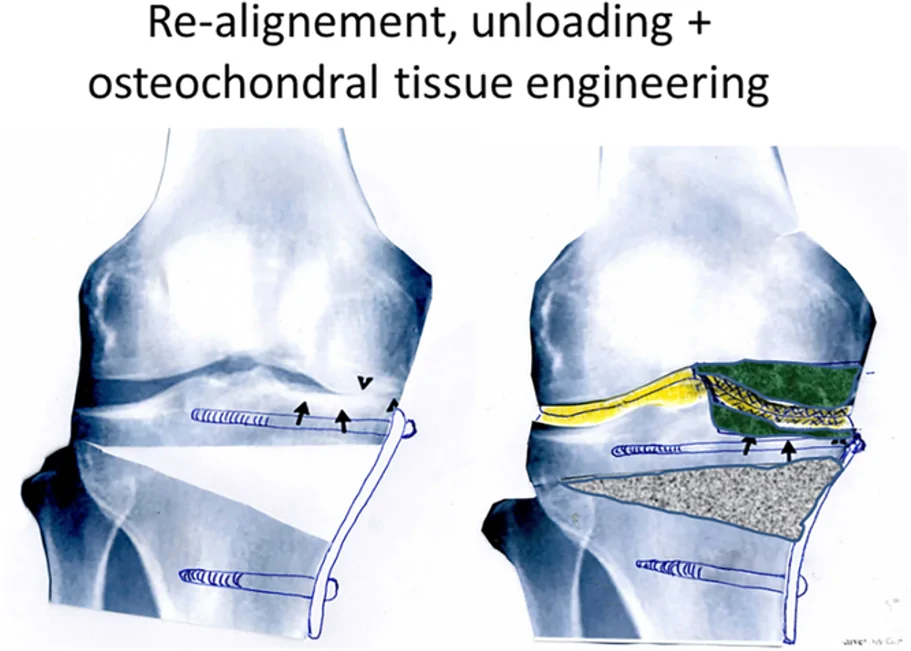
Large resurfacing with biological reconstructions of large osteochondral defects and osteoarthritis (OA) needs to be unloaded for a long time with unloading osteotomies to protect the maturation process.

An unloading osteotomy could be indicated to treat large defects in a joint without any malalignment, small to large defects in a joint with malalignment, and all defects with surrounding poor‐quality cartilage. All patients with chondral or osteochondral injuries should be examined with long weight‐bearing x‐rays for measurement of hip‐knee angle. Furthermore, scintigraphy examination could be useful in order to address possible concomitant metabolic processes when cartilage lesions are retrieved in a degenerative joint [41]. A high uptake may indicate the risk of progress into OA and represents an indication to repair the local defect in combination with osteotomy. For the knee joint, at between 0° and 4° of valgus, the contact pressure is approximately equally distributed between the medial and lateral compartments [44]. This loading situation most closely approximates physiologic loading and represents a suitable choice for patients with isolated chondral defects. To avoid overloading in the lateral compartment in patients with isolated chondral defects and varus malalignment, overcorrection to 8–10° of valgus is generally avoided [44]. Supracondylar femoral varus osteotomy is used if the valgus deformity is more than 12° of valgus or if the tilt of the tibial surface is more than 10°. For large unicompartmental osteochondral reconstructions, with or without associated meniscal transplantation, osteotomies are mandatory in order to unload the tissue‐engineered construct during the long‐lasting maturation process (Figure 5).

Another aspect is the posterior tibial slope, with values of 7°–10° [54]. Changes in the tibial slope can have consequences on ACL insufficiency but may also determine where the cartilage lesion is located. A decrease of posterior tibial slope is recommended for ACL‐deficient and ACL‐reconstructed knees and for isolated posterior femoral condyle and tibial plateau osteochondral defects reducing joint compression forces posteriorly [20, 54]. Instead, an increase in tibial slope is desired for a posterior cruciate ligament (PCL) deficient knee, or to protect PCL reconstruction and isolated anterior cartilage lesions [20, 54].

Patella lesions may also need to be unloaded, and this is done by changing the position of the insertion of the patellar ligament at the tibial tuberosity. Ferguson [19] described that a 1.25 cm elevation of the tibial tuberosity decreased patellofemoral contact forces by 60%–80%, while further elevation provided less benefit. Furthermore, proximal patella lesions, if treated with elevation, will be articulating on more proximal cartilage with the knee flexed. These proximal lesions respond poorly to tibial tubercle anteriorization procedures as the operation causes load shift onto the more proximal patella. Distal lateral patellar cartilage damage does well with anterior medialization alone. Contrarily, total patellar, bipolar and lesions associated with trochlear chondral damage respond poorly to anterior medialization alone. In these cases, good results can be obtained when a cartilage repair procedure is added [49].

With regard to the articular surface loading, it is important to discuss weight loss with patients. Obesity has been associated with prolonged activation of quadriceps and gastrocnemii muscles, which can result in prolonged knee joint contact loading and a risk of predisposition of knee OA development and progression in obese patients [1]. Body mass index has been associated with higher absolute tibiofemoral compression, shear forces, and posterior muscle forces during gait [23]. Such findings may act synergically, together with metabolic factors, to accelerate joint damage, with the OA knees less able to accommodate the high loads. In conclusion, the subchondral unit is important for healthy cartilage and for the success of both chondral and osteochondral injury repair and resurfacing. Biomechanical unloading, temporary or definitive, should always be considered when handling patients with cartilage damage.

Finally, another promising option for the surgical treatment of BMLs is represented by implantable bone shock absorbers. They consist of extra‐capsular devices made of titanium and polycarbonate urethane, which are able to unload the medial compartment of the knee by acting as an extra‐articular load absorber [48]. Recent publications highlighted the good results obtained by bone shock absorbers in the treatment of subchondral insufficiency fractures of the knee [47] and OA [22]. In particular, a prospective comparative study demonstrated the superiority of subcutaneous implantable shock absorbers over high tibial osteotomy in the treatment of symptomatic OA, with clinical superiority documented at even 2 years of follow‐up [13]. Furthermore, the abovementioned publications documented a reduced conversion rate to knee arthroplasty, thus recommending the use of bone shock absorbers as disease‐modifying devices, which have the potential to alter joint derangement progression. The literature addressing this treatment is new and still needs to be fully investigated.

A future alternative unloading technique is a joint distraction with the use of external fixators separating the bones around a joint for a prolonged time, usually 2 months. There are several reports published showing long‐term pain relief in patients with OA and that such a temporary unloading may reverse the tissue degeneration seen in OA [30]. The mechanisms behind such effects have been explained, such as that distraction may relieve mechanical stress on the cartilage, potentially preventing further wear and tear and reducing inflammation [8]. An oscillation seen of synovial fluid pressure during distraction frame walking may be of importance to improve impaired tissue nutrition [30]. Joint distraction could also induce peri‐articular bone changes [30]. The improved joint homoeostasis facilitates the induction of cartilage restoration by activating chondrogenic cells in the synovial tissue and synovial fluid. However, there is still a limited amount of data available regarding joint distraction for knee OA, with small sample sizes in each study [21] and with most studies from the same centre. In the future, large multi‐centre studies comparing joint distraction with unloading osteotomies and artificial joint arthroplasties are needed.

## SYNTHETIC MEDIAL MENISCUS IMPLANT

Conservative non‐operative care or meniscectomies are the most applied treatments for middle‐aged patients with meniscus‐related pain. Neither option is likely to restore the meniscus functionality, optimise the knee load distribution, or reduce subchondral bone stress and eventually, oedema and pain. Thus, at a greater age, more invasive treatments such as joint replacements are used. While meniscal replacement traditionally involves the implantation of allografts, problems related to availability, size matching, and costs limit their use.

Meniscal prostheses developed thus far are principally based on tissue engineering concepts and, as of now, can only be used to treat part of the meniscus. The variability in the body's response to these prostheses and the quality of the tissue formed may make it difficult to obtain satisfying results from scaffold‐type meniscus under extreme knee loading conditions, especially in older patients. Therefore, there is a need to accommodate middle‐aged patients (typically 40–60 years old) in the treatment gap between non‐operative care or meniscus repair and arthroplasty, with a solution that can potentially delay more invasive/reconstructive treatments, preserving the cartilage, reducing pain, and restoring both functionality and normal subchondral pressure.

**Figure 6:**
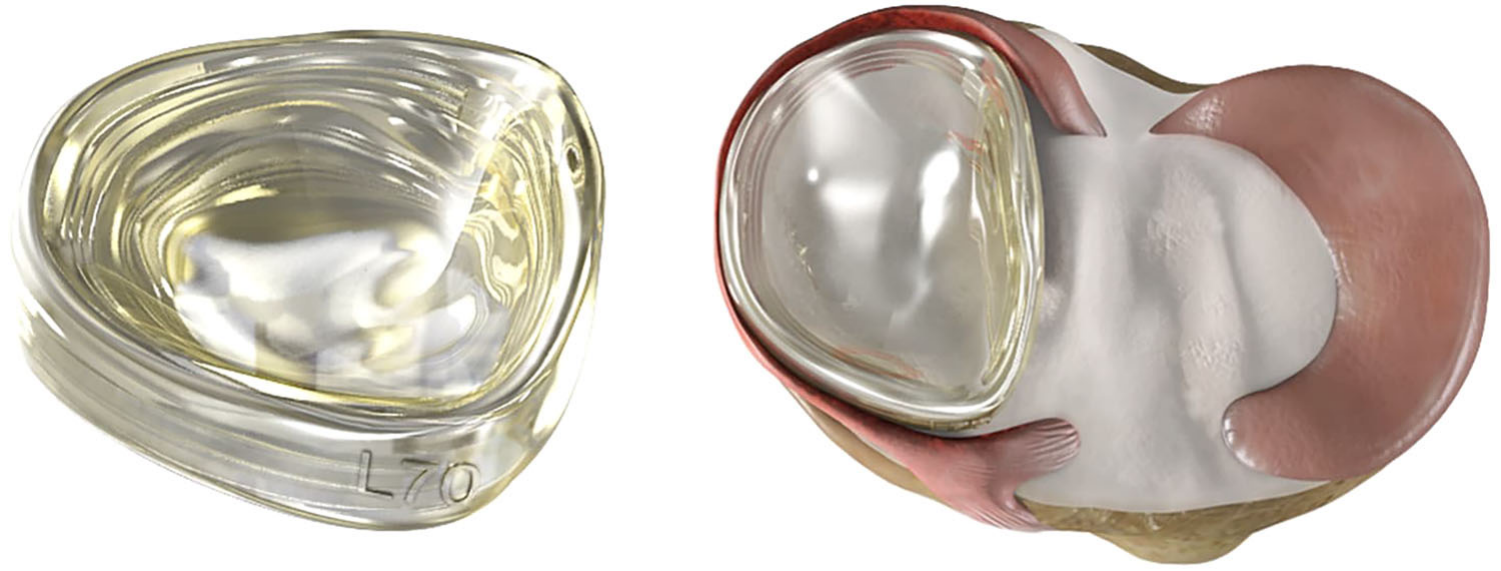
The NUsurface® Implant (left) and its location (top‐view) inside a ‘pocket‐like’ area created using the remaining rim (2–3 mm) of the natural medial meniscus (right).

The NUsurface® Implant (Active Implant LLC, Figure 6) was designed to mimic the shape and function of the natural meniscus without the need to fixate or anchor it. This device is a flexible, self‐centreing, discoid‐shaped composite polymeric implant composed of polycarbonate‐urethane (PCU), reinforced with ultra‐high‐molecular‐weight‐polyethylene (UHMWPE) fibres. A non‐anchored interposition spacer enables straightforward implantation without significant removal of load‐bearing bone while maintaining cartilage and ligament structures.

**Figure 7:**
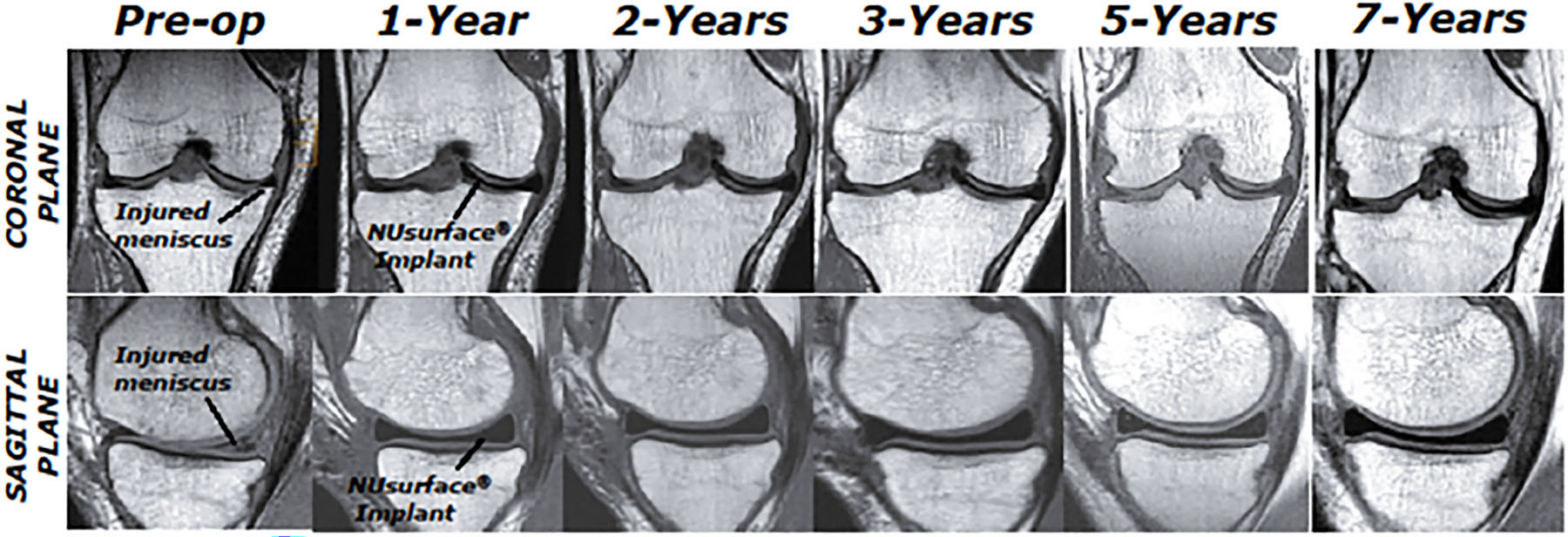
Representative coronal and sagittal MRI of the first NUsurface® patient, a 68‐year‐old male with unstable medial meniscus root tear.

The material properties of this implant were tailored to provide an optimal pressure distribution, and to reduce stress on articular cartilage and subchondral bone. The implant manufacturing process integrates the UHMWPE fibres into a PCU matrix. The final product contains a network of structures designed specifically to help mimic the fibrous structure, function, and mechanical properties of the natural meniscus. The implant's structure was developed based on MRI studies [11, 14], biomechanical studies and cadaver measurements [40, 63], computational analyses [15], wear testing [16], and a pre‐clinical in vivo investigation, in which a quantitative cartilage evaluation was conducted [72]. The device was used in humans in 2008 as a first‐in‐human series. A representative MRI series of the first implanted patient can be found in Figure 7. Significant improvements in patient‐reported outcomes, measured using KOOS and VAS pain scores, were also reported.

Following the first encouraging clinical experience, a multi‐centre randomised clinical trial was conducted, with 1 year of follow‐up versus conservative treatment. The Venus ‘Verifying the Effectiveness of the NUsurface System’ Study had 61 patients implanted versus 66 controls in 10 clinical sites, while the Sun ‘Safety Using NUsurface’ Study had 115 patients implanted without a control group in 13 Clinical sites [71]. Patients in the surgery group showed significant improvement in all subjective outcomes. Specifically, significant improvements were documented in all KOOS subscales. MRIs taken over a 2‐year period indicate that the device maintained the condition of adjacent cartilage in the majority of patients. The most common device‐related adverse events reported during the study were infection, pain, dislocation and failure of the device, with many of these found to be related to either patient selection or surgical technique. Even in cases where the device had to be removed within the 2‐year follow‐up period of the trial, more than 70% of the patients requested another device. Nonetheless, preliminary results coming from the ongoing studies seemed to confirm the beneficial effects documented in the previous publications [42]. The Venus Study was also conducted in Europe and had the same characteristics as the one in the United States. The follow‐up of the European sites exceeded 36 months with the same clinical results related to the improvement of all KOOS subgroups and regarding the preservation of the cartilage of the medial femoral condyle. However, among the complications, there was the need for reoperation in 30% of patients, a condition that was difficult to accept.

To date, synthetic meniscal substitutes are not available on the market. However, the good preliminary results together with the biomechanical studies constitute an important scientific and clinical potential for the development of stable meniscal substitutes with improved wear resistance.

## CONCLUSIONS

The surgical treatment of BMLs is challenging. While good results are reported in the literature for many of the available surgical options, the best approach has not yet been identified. The aim of the interventions described in this paper could be either to stabilise the microtrabecular tissue or to promote a healing response. Subchondroplasty, orthobiologics intralesional injections, as well as unloading osteotomies and meniscal implants are all promising emerging options to address different aetiologies of BMLs. However, the evidence is limited to a few studies analysing a limited number of patients, and larger, high‐quality studies are needed in order to identify the best surgical treatment for BMLs.

## AUTHOR CONTRIBUTIONS

**Giuseppe Filardo**: Conceptualisation; writing—review and editing; supervision. **Luca Andriolo**: Conceptualisation; writing—original draft preparation; writing—review and editing. **Elizaveta Kon**: Conceptualisation; writing—original draft preparation. **Alessandro Sangiorgio**: Writing—original draft preparation. **Mats Brittberg**: Writing—original draft preparation. **Vincenzo Condello**: Writing—original draft preparation. **Jack Farr**: Writing—original draft preparation. **Andrew S. Levy**: Writing—original draft preparation. All authors have read and agreed to the published version of the manuscript.

## CONFLICT OF INTEREST STATEMENT

The authors declare no conflicts of interest.

## ETHICS STATEMENT

No ethical committee approval or patient consent was needed due to the nature of the study.

## Data Availability

Data sharing is not applicable to this article as no data sets were generated or analysed during the current study.
